# The Opiliones tree of life: shedding light on harvestmen relationships through transcriptomics

**DOI:** 10.1098/rspb.2016.2340

**Published:** 2017-02-22

**Authors:** Rosa Fernández, Prashant P. Sharma, Ana Lúcia Tourinho, Gonzalo Giribet

**Affiliations:** 1 Museum of Comparative Zoology, Department of Organismic and Evolutionary Biology, Harvard University, 26 Oxford Street, Cambridge, MA 02138, USA; 2 Department of Zoology, University of Wisconsin-Madison, 352 Birge Hall, 430 Lincoln Drive, Madison, WI 53706, USA; 3 Instituto Nacional de Pesquisas da Amazônia, Coordenação de Biodiversidade (CBIO), Avenida André Araújo, 2936, Aleixo, CEP 69011-970, Manaus, Amazonas, Brazil

**Keywords:** Eupnoi, Dyspnoi, Cyphophthalmi, Laniatores, phylogenomics, Arachnida

## Abstract

Opiliones are iconic arachnids with a Palaeozoic origin and a diversity that reflects ancient biogeographic patterns dating back at least to the times of Pangea. Owing to interest in harvestman diversity, evolution and biogeography, their relationships have been thoroughly studied using morphology and PCR-based Sanger approaches to infer their systematic relationships. More recently, two studies utilized transcriptomics-based phylogenomics to explore their basal relationships and diversification, but sampling was limiting for understanding deep evolutionary patterns, as they lacked good taxon representation at the family level. Here, we analysed a set of the 14 existing transcriptomes with 40 additional ones generated for this study, representing approximately 80% of the extant familial diversity in Opiliones. Our phylogenetic analyses, including a set of data matrices with different gene occupancy and evolutionary rates, and using a multitude of methods correcting for a diversity of factors affecting phylogenomic data matrices, provide a robust and stable Opiliones tree of life, where most families and higher taxa are precisely placed. Our dating analyses using alternative calibration points, methods and analytical parameters provide well-resolved old divergences, consistent with ancient regionalization in Pangea in some groups, and Pangean vicariance in others. The integration of state-of-the-art molecular techniques and analyses, together with the broadest taxonomic sampling to date presented in a phylogenomic study of harvestmen, provide new insights into harvestmen interrelationships, as well as an overview of the general biogeographic patterns of this ancient arthropod group.

## 1. Introduction

Opiliones (‘harvestmen’ or ‘daddy longlegs’) are a remarkable group of arachnids (electronic supplementary material, figure S1), with a fossil record dating to the Early Devonian, having diversified in its main lineages by the Carboniferous [1–3], and showing ancient vicariant patterns that accord with their modern distribution [4–8]. They show fascinating reproductive behaviours, including paternal and biparental care [9–12], and constitute an example of the first direct transfer of sperm on land via a penis [1].

The phylogeny of the order Opiliones and its four extant suborders—Cyphophthalmi (the mite harvestmen), Eupnoi (the daddy longlegs), Dyspnoi (the ornate harvestmen) and Laniatores (the armoured harvestmen)—has received considerable attention, based on morphological [13–17], molecular [18–21] and combined datasets [3,22,23]. After some debate, the relationships among the Opiliones suborders have been settled, with Cyphophthalmi constituting the sister group of Phalangida, the latter divided in Palpatores (Eupnoi + Dyspnoi) and Laniatores. More recently, a few studies have used phylogenomic data derived from transcriptomes to further test relationships among Opiliones [24–26], but these pioneering studies included a handful of species (8–14) representing just a few families. Likewise, the internal relationships of each of the four suborders have received attention, mostly using molecular [4,20,27–29] and combined analyses of morphology and molecules [8]. Other morphological analyses have focused on particular suborders [30–33]. Recently, a Dyspnoi cladogram was proposed based on a summary of proposed relationships [34]. In addition, dozens of papers have explored the relationships of individual families or groups of closely related species.

While many aspects of the phylogeny of Opiliones are now well understood, a few remain largely unresolved or understudied. For example, within Cyphophthalmi, the relationships among its six families, and even the monophyly of Sironidae, remain unsettled [8]. Relationships within Eupnoi—the group that includes the true ‘daddy longlegs’—are barely explored from a molecular perspective [20,35,36], and no study has included all the relevant diversity. Resolution within these clades is poor, with the exception of the deepest division between Caddoidea and Phalangioidea [20]. Relationships within Dyspnoi are just beginning to settle [28,29,37], but, for example, only recently was it recognized that Acropsopilionidae are related to Dyspnoi and not to Eupnoi [20], based on a handful of Sanger-sequenced molecular markers. This resulted in transferring a clade of Opiliones from Eupnoi to Dyspnoi, as the sister group to all other members (Ischyropsalidoidea + Troguloidea), and therefore deserves further testing using a modern and more complete dataset. Finally, relationships within Laniatores have changed considerably after the study of Sharma & Giribet [27], as the taxonomy of this large clade of Opiliones has been in flux, with description of several families in recent years [27,38–40]. Some novel results include the proposal of a sister group relationship of the New Zealand endemic family Synthetonychiidae to all other Laniatores [19,27]—a result that hinged on partial data from a single species. In addition, the relationships among many families remain unstable.

Recent application of dense taxon sampling using large numbers of genes through modern phylogenomic approaches (e.g. based on genome and Illumina-based datasets) has resolved family-level relationships of a diversity of groups of arachnids [41–44] and other arthropods [45,46]. We applied these methodologies to Opiliones phylogenetics to produce a densely sampled family-level phylogeny by analysing 54 harvestman transcriptomes (40 newly generated for this study and 14 previously published) representing 40 of the 50 currently recognized extant families (80% familial representation).

## 2. Material and methods

### (a) Specimens

Specimens of Opiliones selected for this study were preserved in RNA*later* and transferred to liquid nitrogen upon arrival to the laboratory, or flash-frozen, and subsequently stored at −80°C. Total RNA, mRNA purification and library construction protocols are explained in detailed in the electronic supplementary material, Extended material and methods and S1).

Our final matrix comprises 54 taxa, including 10 Cyphophthalmi (four families included; Ogoveidae and Troglosironidae missing), nine Eupnoi (representatives of all five families included), nine Dyspnoi (all eight families included), and 26 Laniatores (representatives of 23 families included; missing Gerdesiidae, Guasiniidae, Icaleptidae, Kimulidae, Metasarcidae, Nippononychidae, Pyramidopidae and Tithaeidae, all families of low diversity and relatively narrow distribution in places difficult to access). As outgroups, we included several chelicerates (see electronic supplementary material, Extended material and methods and table S1).

All raw sequences are deposited in the SRA archive of GenBank under accession numbers specified in electronic supplementary material, table S1. Data on specimens are available from MCZbase (http://mczbase.mcz.harvard.edu).

### (b) Orthology assignment and phylogenetic analyses

Orthology assignment was based on the OMA algorithm v. 0.99.z3 [47], as specified in detail in our previous work [48]. Multiple sequence alignment, alignment masking and criteria for matrix construction followed our previous workflows [48] and are detailed in the electronic supplementary material. Maximum-likelihood inference was conducted with PhyML-PCMA [49], ExaML [50] and PhyML v. 3.0.3. Bayesian analyses were conducted with ExaBayes [51] and PhyloBayes MPI 1.4e [52] using the site-heterogeneous CAT-GTR model of evolution in the latter software [53]. Compositional homogeneity of each gene and taxon was evaluated in BaCoCa [54]. Details on the different matrices, priors and heuristics are catalogued in the electronic supplementary material.

### (c) Molecular dating

The fossil record of Opiliones is well documented, and most key fossils have been included in prior phylogenetic analyses, making their placement in a phylogenetic context precise. We mostly follow the strategy and fossil placement of Sharma & Giribet [25], who conducted tip dating in one of their analyses. Exact details about the fossils selected and the type of constraints used are described in the electronic supplementary material, Extended material and methods.

Divergence dates were estimated using the Bayesian relaxed molecular clock approach as implemented in PhyloBayes v. 3.3f [52] under the autocorrelated lognormal and uncorrelated gamma multipliers models, resulting in four analyses (i.e. these two models were applied to both calibration configurations described above, with the age of *Eophalangium* as the minimum age of Cyphophthalmi or as the floor of Opiliones). Two independent MCMC chains were run for each analysis (10 000–12 000 cycles). The calibration constraints were used with soft bounds [55] under a birth–death prior.

## 3. Results and discussion

**Figure 1.**
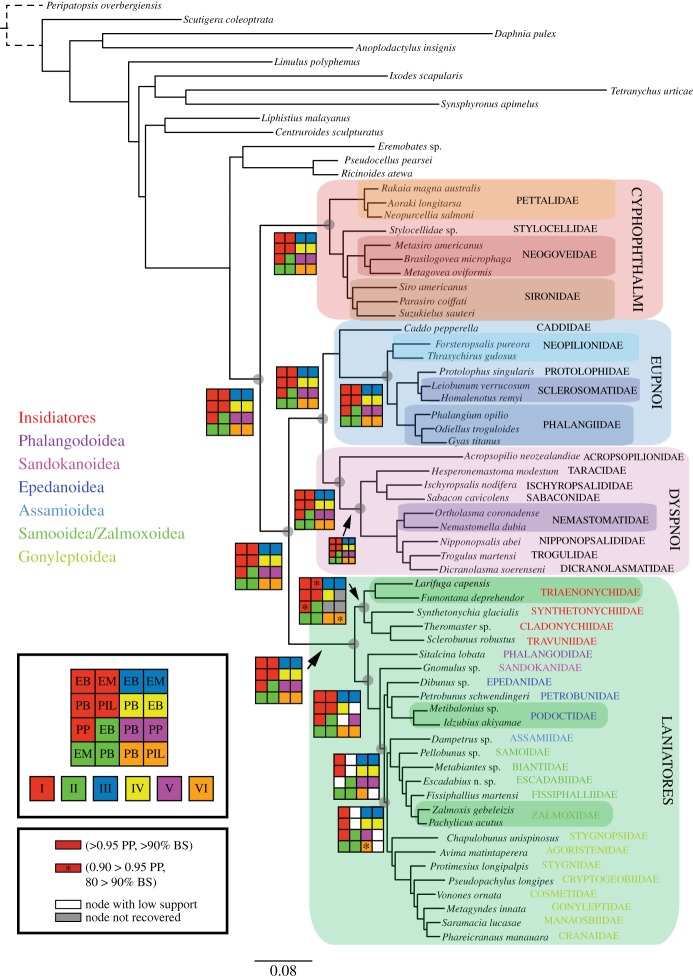
Phylogenetic hypothesis based on the 78-gene matrix I analysed in PhyML_PCMA (−*ln*L = −248960.37) Selected deep nodes (grey circle) show Navajo rug illustrating support under specific data matrices and analyses. In Laniatores, coloured text for family names indicates superfamily boundaries. EB: ExaBayes. EM: ExaML. PB: PhyloBayes. PIL: PhyML with integrated branch lengths. PP: PhyML-PCMA. (Online version in colour.)

All results are based on three original data matrices of 78 genes (matrix I; more than 90% gene occupancy), 305 genes (matrix II; more than 75% gene occupancy) and 1550 genes (matrix III; more than 50% gene occupancy), as well as subsets of these matrices (see Material and methods). Figure 1 (see also electronic supplementary material, figure S2) illustrates the topology obtained for matrix I in PhyML_PCMA, with a Navajo rug representing the support for the 16 analyses conducted for the different matrices and methods.

### (a) Higher-level Opiliones phylogenetics

Our analyses recover a stable relationship among the four extant Opiliones suborders, each well supported as monophyletic in all the analyses (figure 1), as consistently found in a variety of published Opiliones analyses (e.g. [16,18,19,20,24–26]), including phylogenomic ones [24–26]. Likewise, we found Cyphophthalmi as sister group to Phalangida, monophyly of Palpatores, and a sister group relationship of Palpatores to Laniatores, as in nearly all recent studies cited above. However, most published analyses found little support for the resolution within each suborder—in Sanger-based analyses due to insufficient sequence data and in phylogenomic analyses due to few taxa. The resolved relationships within each of the four suborders are thus the most novel aspects of this study. Each suborder is therefore discussed in detail below.

### (b) Cyphophthalmi—the mite harvestmen

**Figure 2.**
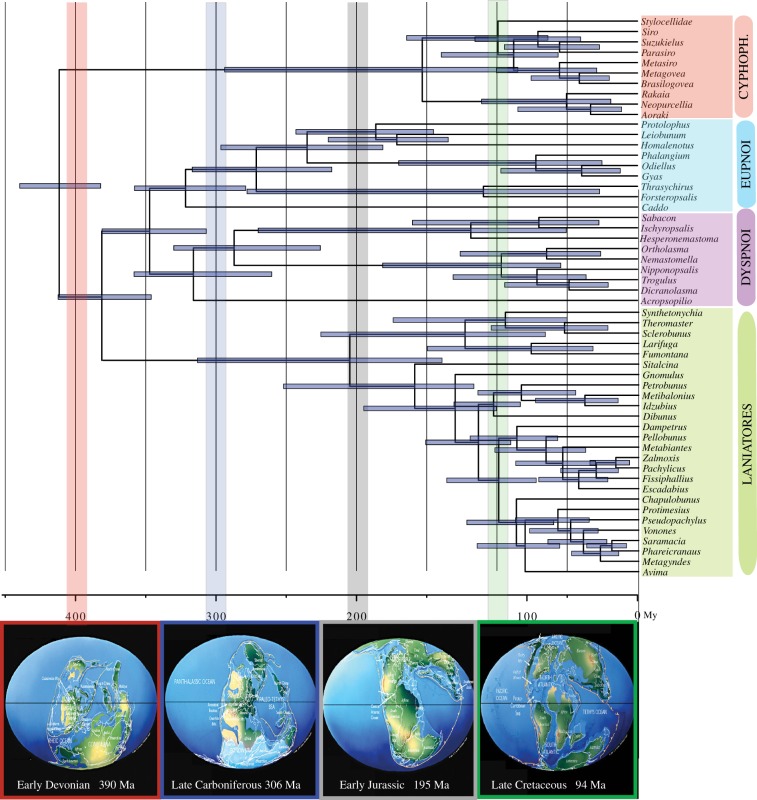
(*a*) Chronogram of Opiliones evolution for the 78-gene dataset with 95% highest posterior density (HPD) values for the dating for the first calibration configuration (i.e. the age of *Eophalangium* as the minimum age of Cyphophthalmi) under uncorrelated gamma model. Down, palaeogeographical reconstruction according to Christopher R. Scotese (maps modified from http://www.scotese.com/earth.htm) at some of the key ages of the split of Opiliones main lineages, as recovered by the molecular dating analysis. Vertical bars indicate correspondence with each palaeomap following a colour code. (Online version in colour.)

The members of the suborder Cyphophthalmi (electronic supplementary material, figure S1*a*) have received special attention phylogenetically due to their antiquity, their global distribution and their low vagility (e.g. [4,8]). Here, we confirm the division of Cyphophthalmi into the temperate Gondwanan family Pettalidae and the remaining families (Stylocellidae, Neogoveidae, Sironidae) (figure 1), a divergence that took place around the Jurassic, diversifying during the Cretaceous (figure 2). While the New Caledonian endemic Troglosironidae and the west African endemic Ogoveidae were not included, their phylogenetic affinity to Neogoveidae in the clade Sternophthalmi is strongly supported by an array of morphological and molecular datasets [8].

Relationships among Stylocellidae, Neogoveidae and Sironidae are unstable, and two topologies prevail: (Stylocellidae, (Neogoveidae, Sironidae)) and (Sironidae, (Stylocellidae, Neogoveidae)), neither topology supporting the taxon Boreophtahlmi, grouping Stylocellidae and Sironidae [8] (figure 1; electronic supplementary material, figure S2). The first topology is preferred by the most complete dataset. However, in some of the analyses using fewer genes the Stylocellidae species nests within Sironidae, albeit without support. These two alternatives will require further examination with more stylocellid samples, as the alternative topologies may also have an impact on the dating, which suggests an initial diversification around the Cretaceous (figure 2).

Monophyly of Neogoveidae is recovered in all analyses, with the exception of the PhyloBayes analysis of the 78-gene matrix (electronic supplementary material, figure S2). The placement of the North American *Metasiro* with the typical Neotropical neogoveids corroborates previous molecular hypotheses of the delimitation of this clade [8,56].

Monophyly of Sironidae, here represented by three genera of the three main lineages of this Laurasian family (*Siro*, *Parasiro* and *Suzukielus*), is unstable across analyses (electronic supplementary material, figure S2). Monophyly of Sironidae has been difficult to obtain in molecular analyses as well as with morphology due to differences in family-level characters in the western Mediterranean *Parasiro* and the Japanese *Suzukielus* [8].

### (c) Eupnoi—the daddy longlegs

Family-level Eupnoi phylogenies are scarce [8,20,35] and have typically undersampled Southern Hemisphere lineages. Our analyses support the well-known division of Caddoidea (electronic supplementary material, figure S1*b*) and Phalangioidea (electronic supplementary material, figure S1*c*,*d*), which in turn divides into the Southern Hemisphere Neopilionidae and the mostly Northern Hemisphere families Phalangiidae, Sclerosomatidae and Protolophidae—although Phalangiidae and Sclerosomatidae have later diversified in the Southern Hemisphere (figure 1; electronic supplementary material, figure S2). The sister group relationship among Protolophidae and Sclerosomatidae has been found in previous analyses [20,35], and in fact some have considered Protolophidae a junior synonym of Sclerosomatidae [57]. However, resolution among the families of Phalangioidea has received little or no support in previous studies. Our results thus provide, for the first time, a well-resolved Eupnoi phylogeny, including the placement of *Gyas titanus* within Phalangiidae, as suggested by Hedin *et al*. [35], instead of within Sclerosomatidae. We were not able to include any members of the phylogenetically unstable ‘*Metopilio* group’ [19,35]. We thus find Phalangioidea divided into three main clades: Neopilionidae, Sclerosomatidae/Protolophidae and Phalangiidae (including *Gyas*). However, the systematics of this large group of Opiliones, with nearly 200 genera and 1800 species, will require much denser sampling before the group can be properly revised.

### (d) Dyspnoi—the ornate harvestmen

The global phylogeny of Dyspnoi has received attention from different workers using morphology and molecules, but only recently there has been modern treatment. Groh & Giribet [20] finally circumscribed the suborder, transferring Acropsopilionidae from Eupnoi to the sister group of all other Dyspnoi based on molecular data analyses of a few Sanger-sequencing genes and morphological examination. Our phylogenomic datasets corroborate this topology (figure 1), placing *Acropsopilio neozealandiae* as the sister group to the other Dyspnoi, with the monophyly of each of Ischyropsalidoidea and Troguloidea being fully supported. While the position of the Cretaceous fossil *Halitherses grimaldii* remains uncertain, their large eyes (resembling those of caddids and acropsopilionids) and their troguloid facies [58] suggest a phylogenetic placement between Acropsopilionoidea and the remaining Dyspnoi [59], perhaps as sister group to Troguloidea or to Troguloidea + Ischyropsalidoidea. However, the ‘caddoid’ gestalt is now known from Caddoidea, Phalangioidea (in the members of the genus *Hesperopilio*; see [20]) and Acropsopilionoidea, and enlarged eyes are thus best optimized as a symplesiomorphy of Palpatores.

### (e) Laniatores—the armored harvestmen

The phylogeny of Laniatores—the largest suborder of Opiliones with more than 4200 described species—has received recent attention at many levels [19,27,60,61]. In an unpublished thesis, Kury [62] divided Laniatores into Insidiatores (electronic supplementary material, figure S1*h*–*i*) and Grassatores (electronic supplementary material, figure S1*j*–*p*), a division found here, but not in other studies that included a meaningful sampling of Laniatores [19,27]. These found the New Zealand endemic Synthetonychiidae to be sister group to all other Laniatores (Eulaniatores *sensu* Kury [63]). Of special interest also was the phylogenetic position of the unstable North American *Fumontana deprehendor*, a member of the mostly temperate Gondwanan family Triaenonychidae that was poorly resolved in prior studies. Our analyses do find a sister group relationship of *Fumontana* to the representative of the Southern Hemisphere Triaenonychidae in virtually all analyses, with a Cretaceous divergence (figure 2). *Synthetonychia* is either sister group to the represented travunioids in most analyses (except for matrices IV and V; see electronic supplementary material, figure S2) or sister group to Triaenonychidae, as originally proposed by Forster [64]. Further discussion on Insidiatores will require increased diversity of genera both within Triaenonychidae and within the travunioid families (see for example [57]).

Resolution within Grassatores has remained elusive except for the recognition of a main division between Phalangodidae and the remaining Grassatores and of the superfamilies Gonyleptoidea, Assamioidea, Zalmoxoidea and Samooidea, and perhaps a clade of southeast Asian families, Epedanoidea [27]. Some of these clades were not supported in re-analyses of the Sharma & Giribet dataset [60,61]. Here, we consistently find Phalangodidae (represented by *Sitalcina lobata*) to be the sister group of the remaining Grassatores.

The southeast Asian endemic Sandokanidae [65] is resolved as the sister group to the remaining families, a clade supported by nearly all matrices and most analyses (only some analyses find this clade without support) (figure 1). The position of Sandokanidae has been difficult to resolve in prior analyses [19,27], which sometimes suggested a relationship to Epedanoidea. Here, we reject this hypothesis and support Sandokanidae as the second offshoot of the Grassatores, contradicting earlier hypotheses dividing Grassatores in Oncopodoidea versus Gonyleptoidea (e.g. [13,15]).

The sister group of Sandokanidae divides into the largely southeast Asian Epedanoidea (represented here by members of Epedanidae, Petrobunidae and Podoctidae) and a clade including Assamioidea, Samooidea, Zalmoxoidea and Gonyleptoidea, this divergence being Cretaceous (figure 2; electronic supplementary material, figure S3). This coincides with the first Laniatores fossils, which were already present in the terranes of today's Myanmar [66]. Epedanoidea is monophyletic in all analyses (sometimes without significant support), except for the PhyloBayes analysis of matrix VI (electronic supplementary material, figure S2), and it is resolved with Epedanidae being sister group to a clade of Petrobunidae and Podoctidae (electronic supplementary material, figure S2; see also [61]). Resolving this may require additional taxa, including the missing family Tithaeidae. Epedanoidea has however been difficult to recover in a recent analysis focusing on Podoctidae [61].

The sister group of Epedanoidea, a clade composed of Assamioidea–Zalmoxoidea–Samooidea–Gonyleptoidea, is well supported in virtually all analyses (figure 1). Internal resolution among these superfamilies had found conflict in prior studies [19,27], as it probably required additional molecular data to resolve this rapid radiation of Laniatores families. Phylogenomic data find the much-needed information to resolve this clade, here assigned a Cretaceous age (figure 2; electronic supplementary material, figure S3).

A sister group relationship of *Dampetrus* (an assamiid; the only representative of Assamioidea included here) to Zalmoxoidea–Samooidea is found with all data matrices except V and VI, but does not receive support in most analyses with matrix I. Fewer genes seem to be necessary to support fully a clade of Zalmoxoidea and Samooidea, which was found in prior Sanger-based studies [27]. However, Samooidea is paraphyletic with respect to Zalmoxoidea in about half of the analyses (electronic supplementary material, figure S1), although in others they are reciprocally monophyletic. The *bona fide* samooid *Pellobunus* from Panama is sister group to the remaining members of this clade, followed by a representative of Biantidae, *Metabiantes* from South Africa, and by a clade of Zalmoxoidea, including an Amazonian specimen we tentatively placed in the genus *Escadabius* (Escadabiidae), and then the representatives of Fissiphalliidae (*Fissiphallius martensi*) and Zalmoxidae (*Zalmoxis*, *Pachylicus*). Except for the clade placing *Metabiantes* with the zalmoxoids, relationships within this clade are stable (electronic supplementary material, figure S2). Further samooid and zalmoxoid missing families (Kimulidae, Stygnommatidae, Guasiniidae and Icaleptidae; all exclusively Neotropical) should be sampled to resolve the issue of the reciprocal monophyly of the families.

**Figure 3.**
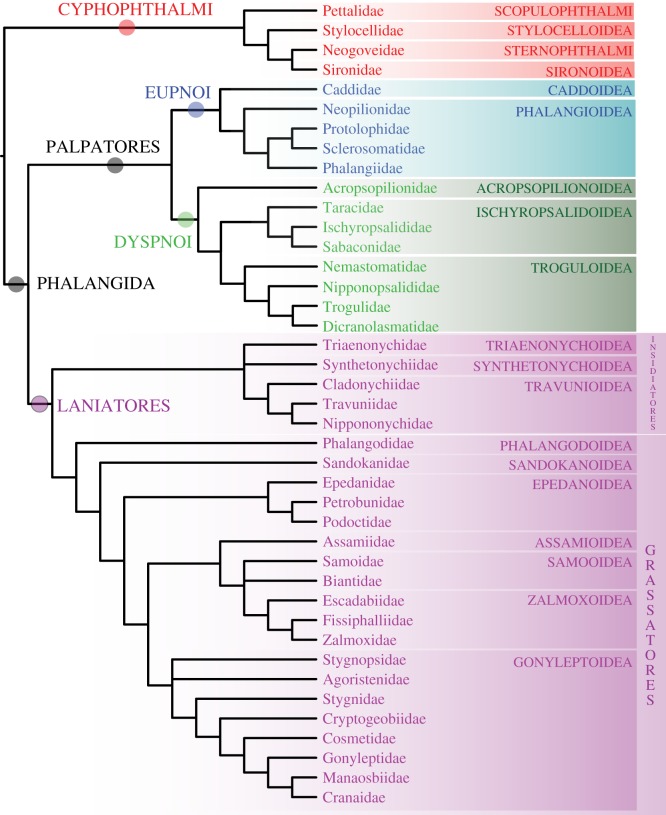
Summary tree of familial and superfamilial relationships of Opiliones supported in this study, with major nodes highlighted. (Online version in colour.)

Gonyleptoidea is restricted to an expanded Neotropics (some gonyleptid species make it into Patagonia and some cosmetids quite for north into the USA). Stygnopsidae (*Chapulobunus*) is sometimes sister group to all other gonyleptoids, followed by Agoristenidae (*Avima*), although the position of Agoristenidae is not well resolved. Agoristenids had been proposed as the sister group of the non-stygnopsid gonyleptoids in previous analyses [27], as shown here in some trees (figures 1–3), but most matrices suggest a sister group relationship of Agoristenidae and Stygnopsidae. Stygnidae (*Protimesius*) is well supported as sister group to all the remaining families, followed in a ladder-like fashion by the families Cryptogeobiidae (*Pseudopachylus*), Cosmetidae (*Vonones*), Gonyleptidae (*Metagyndes*), Manaosbiidae (*Saramacia*) and Cranaidae (*Phareicranaus*). This topology is fully compatible with more detailed recent analyses of Gonyleptoidea [39,40,67], some of which consider Manaosbiidae and Cranaidae subfamilies of Gonyleptidae, as originally proposed by Roewer (see [67]), although this was not accepted in subsequent studies [39,68]. We thus support a clade including these three families, which has also been called Greater Gonyleptidae (GG) [39,68].

### (f) Molecular dating

The molecular dating analyses for both calibration configurations (i.e. the age of *Eophalangium* as the floor of Opiliones or Cyphophthalmi) yielded very similar results, with the main differences obtained between the autocorrelated and uncorrelated model analyses within each calibration configuration (electronic supplementary material, figure S3). For purposes of conservatism, we discuss results based on the chronogram under the uncorrelated gamma multipliers model using the age of *Eophalangium* as the minimum age of Cyphophthalmi (figure 2), but some of the divergence dates may vary substantially.

Opiliones have often been used as examples of animals with ancient and conservative biogeographic patterns, therefore suitable for vicariance biogeographic analyses [5,19]. One general pattern observed here is a division between temperate Gondwana (the terranes that were once directly connected to Antarctica) and the remaining landmasses, including, in some cases, clades currently in tropical Gondwana. For example, this is the case for Cyphophthalmi, with a main division between the strictly temperate Gondwanan family Pettalidae and the remaining families (this including Laurasian and tropical Gondwanan clades), or in Dyspnoi, with Acropsopilionidae, being mostly distributed in temperate Gondwana, as the sister group to the rest of the Dyspnoi families, restricted to the Northern Hemisphere. Within Eupnoi, Caddidae is mostly Laurasian, but Phalangioidea once more divides into Neopilionidae, restricted to temperate Gondwana (with the exception of *Thrasychiroides*, which extends to the Atlantic rainforest [69]), and the remaining families, mostly Laurasian, although some secondarily extending southwards. Once more, Insidiatores, although somehow unresolved, finds a division between the predominantly temperate Gondwanan family Triaenonychidae (or Triaenonychidae + Synthetonychiidae) and the Northern Hemisphere Insidiatores (Travunioidea). In addition, Triaenonychidae has a basal split between the Northern Hemisphere *Fumontana* and the temperate Gondwanan clade (although here it is represented by a single species), as shown in other published phylogenies of Laniatores [27]. Laniatores depict several other interesting patterns, including two clades of southeast Asian families, Sandokanidae and Epedanoidea, while the remaining species mostly appear to be of Tropical Gondwanan origins, with some remarkable cases of range expansions (e.g. trans-continental disjunctions in Assamiidae, Biantidae, Podoctidae, Pyramidopidae and Zalmoxidae [27,60,70]).

Interestingly, the splits between temperate Gondwana and the rest precede the breakup of Pangea (figure 2), suggesting ancient regionalization across Pangea, as shown in other groups of terrestrial invertebrates [71] and in the early diversification of amphibians [72]. Splits between tropical Gondwana and Laurasia, both in Cyphophthalmi and in Grassatores, seem to be much younger, and may be associated with the breakup of Pangea, possibly representing true Gondwanan/Laurasian vicariant events, and not the result of ancient cladogenesis and Pangean regionalization. Detailed analyses with a much denser sampling within each family should allow further scrutiny of these suggestive distributions.

## 4. Conclusion

Our analysis of a large number of novel transcriptomes has allowed us to propose a stable phylogeny of Opiliones (figure 3). Such analyses of large data matrices have allowed us to place all superfamilies of Opiliones (and 80% of the families) in a resolved phylogenetic context, with only a few spots to be sorted out in areas of the tree where sampling was still limited. Our trees support most traditional relationships within Opiliones and resolve some recalcitrant familial relationships, such as a well-resolved Eupnoi phylogeny, the rejection of Boreophthalmi, the monophyly of Insidiatores and the placement of Stygnopsidae as the most basal family of Gonyleptoidea, among others. We also show that Opiliones exhibit some splits reflecting ancestral Pangean regionalization, whereas others conform with high fidelity to the sequence of Pangean fragmentation, therefore constituting ideal model systems to understand ancient biogeographic patterns.

## Supplementary Material

Figure S1

## Supplementary Material

Figure S2

## Supplementary Material

Figure S3

## Supplementary Material

Table S1

## Supplementary Material

Table S2

## Supplementary Material

Extended Material and Methods

## Data accessibility

Electronic supplementary material, figures S1–S3, tables S1 and S2, and Extended material and methods are available from https://dx.doi.org/10.6084/m9.figshare.c.3691975. All matrices are available at the Dryad Digital Repository: http://dx.doi.org/10.5061/dryad.fs8kv [73].
