# Supplementary figures and images for: The Opiliones tree of life: shedding light on harvestmen relationships through transcriptomics
Source: Proc Biol Sci. 2017 Feb 22;284(1849):20162340. doi: 10.1098/rspb.2016.2340 (PMC5326524; doi:10.1098/rspb.2016.2340)

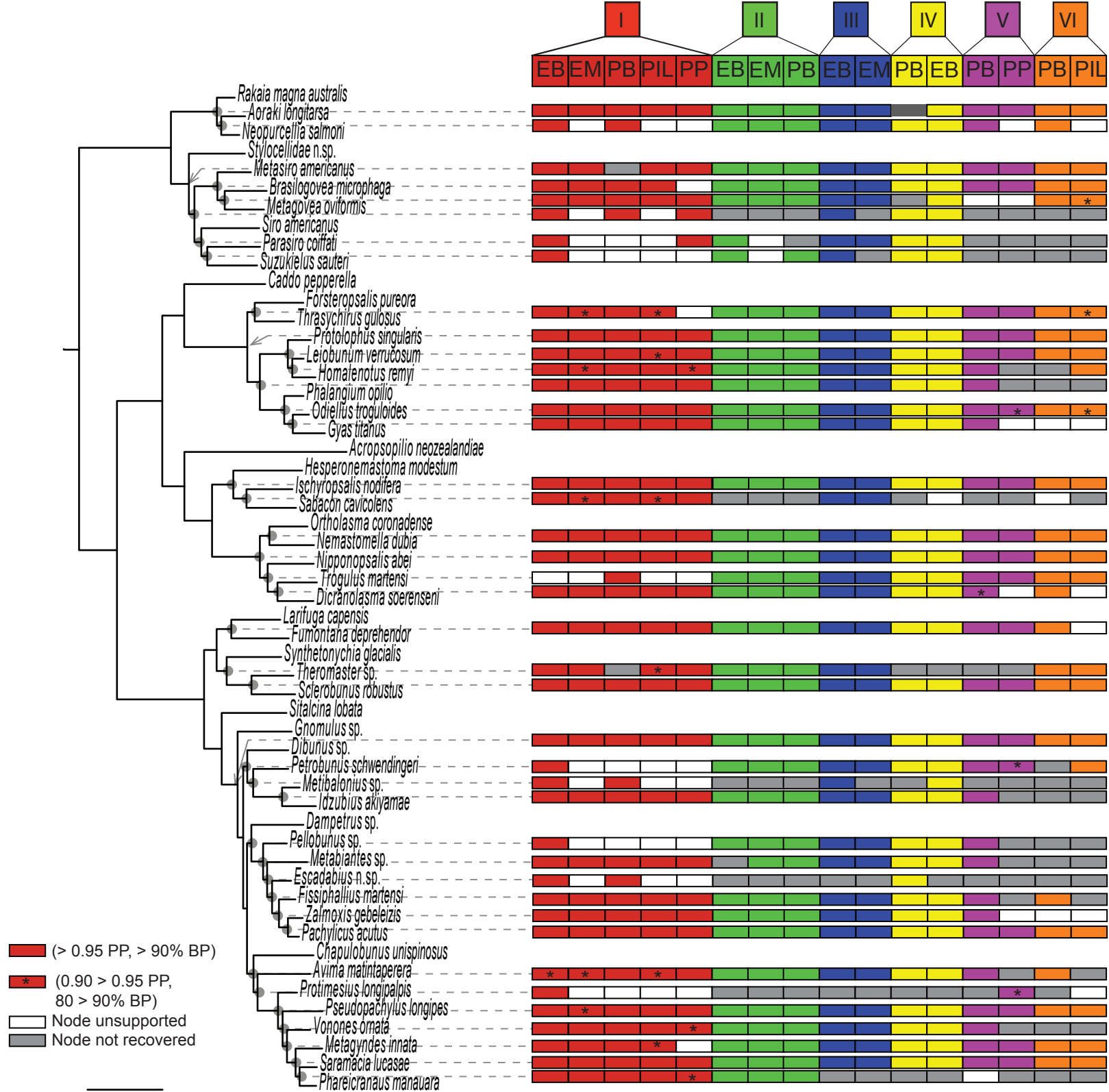

Supplement: Figure S1 [file rspb20162340supp1.pdf]

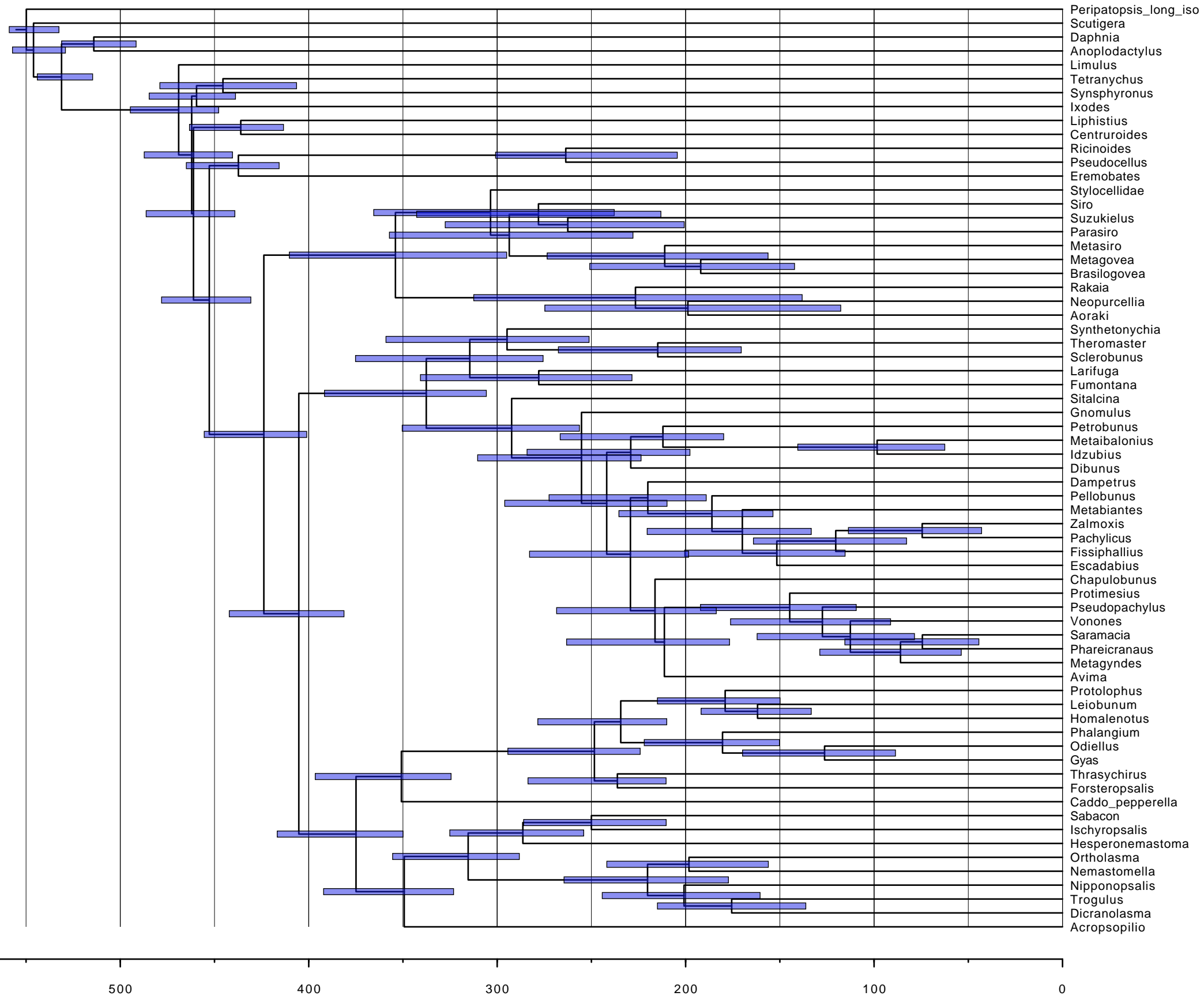

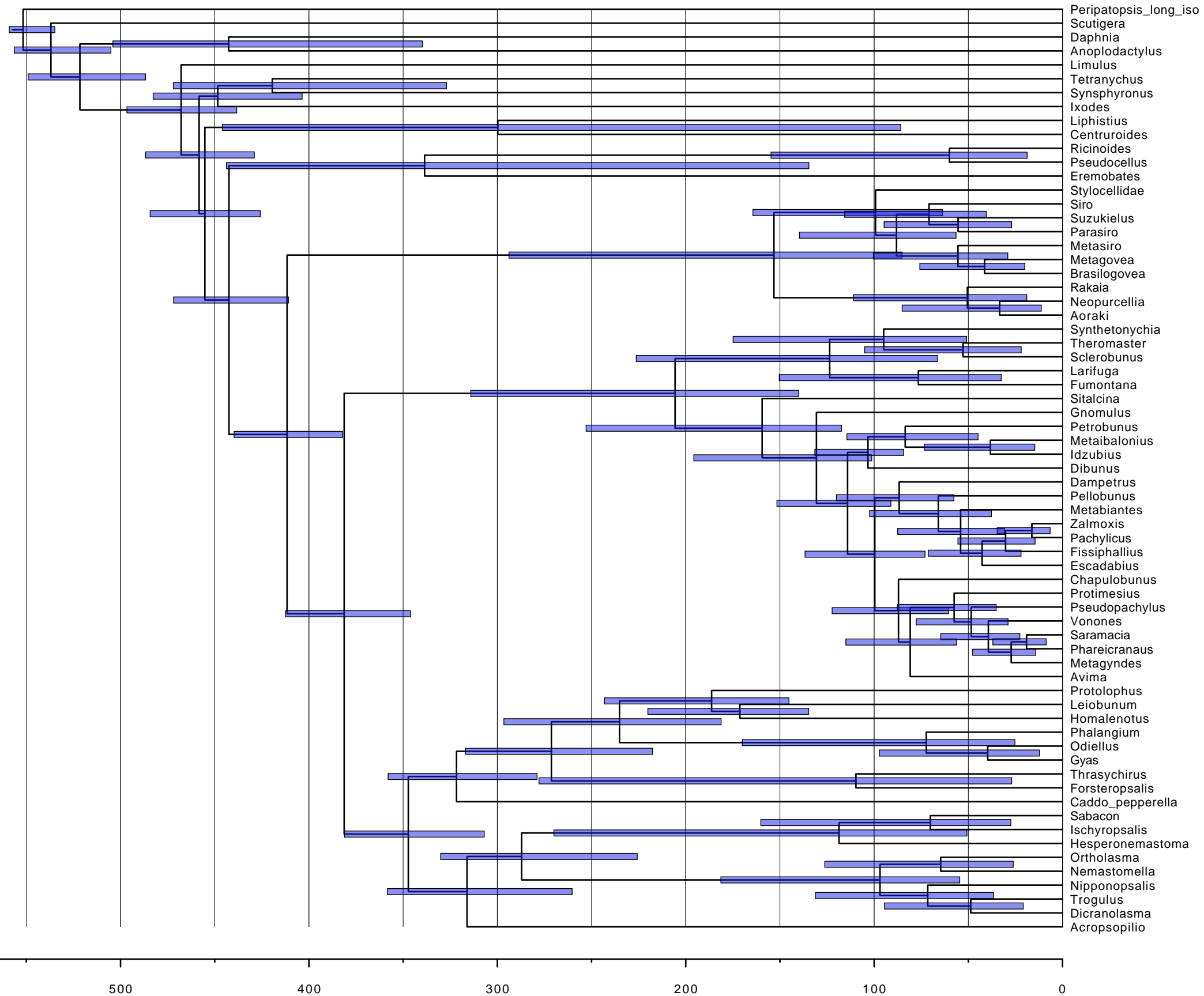

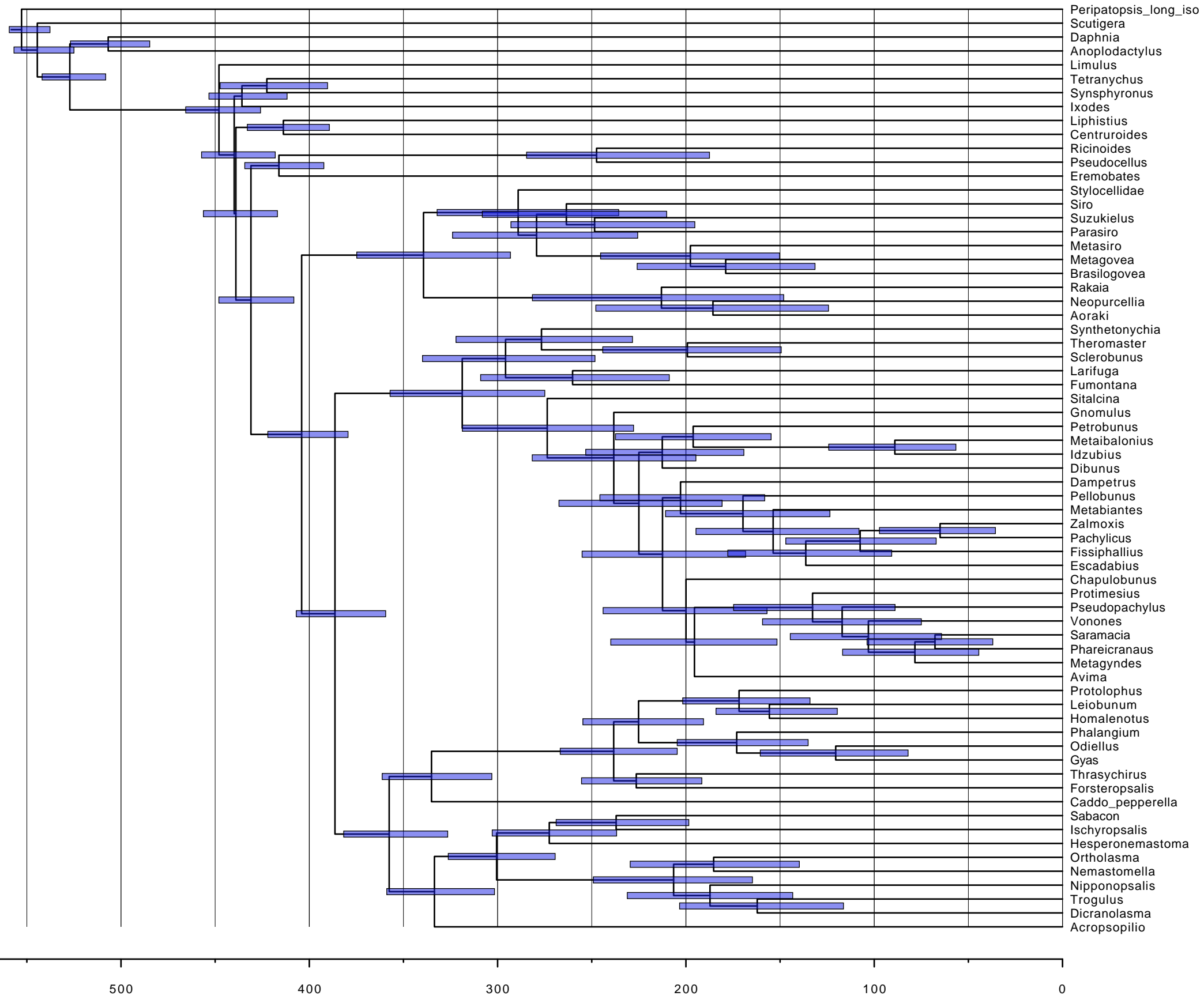

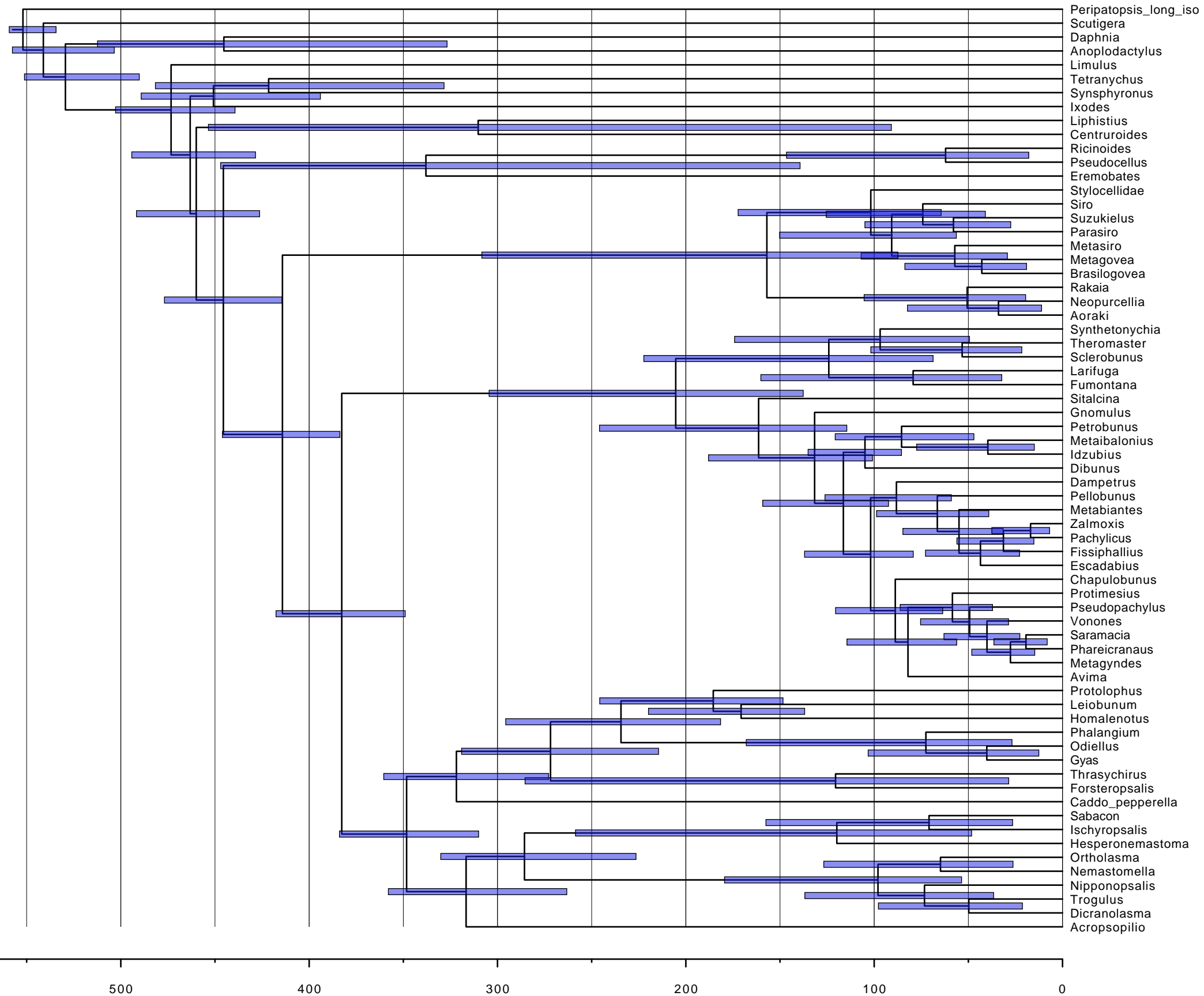

Supplement: Figure S3 [file rspb20162340supp3.pdf]
