# Supplementary material for: The Opiliones tree of life: shedding light on harvestmen relationships through transcriptomics
Source: Proc Biol Sci. 2017 Feb 22;284(1849):20162340. doi: 10.1098/rspb.2016.2340 (PMC5326524; doi:10.1098/rspb.2016.2340)
Supplement: Figure S2 [file rspb20162340supp2.pdf]

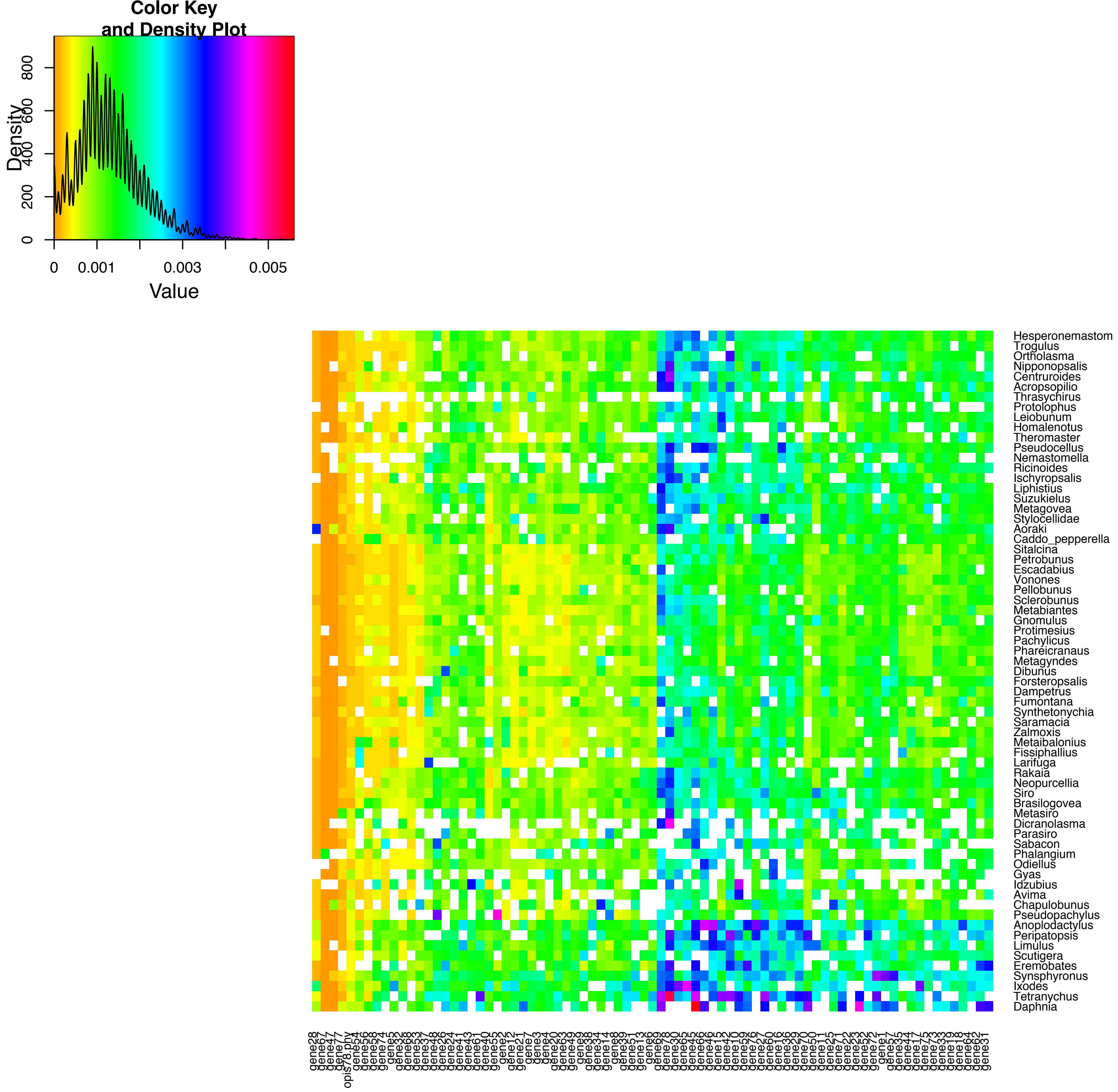
Color Key
and Density Plot
Density
0
200
400
600
800
Value
0
0.001
0.003
0.005
Hesperonemastom
Trogulus
Ortholasma
Nipponopsalis
Centruroides
Acropsopilio
Thrasychirus
Protolophus
Leiobunum
Homalenotus
Theromaster
Pseudocellus
Nemastomella
Ricinoides
Ischyropsalis
Liphistius
Suzukielus
Metagovea
Stylocellidae
Aoraki
Caddo_pepperella
Sitalcina
Petrobunus
Escadabius
Vonones
Pellobunus
Sclerobunus
Metabiantes
Gnomulus
Protimesius
Pachylicus
Phareicranaus
Metagyndes
Dibunus
Forsteropsalis
Dampetrus
Fumontana
Synthetonychia
Saramacia
Zalmoxis
Metaibalonius
Fissiphallius
Larifuga
Rakaia
Neopurcellia
Siro
Brasilogovea
Metasiro
Dicranolasma
Parasiro
Sabacon
Phalangium
Odiellus
Gyas
Idzubius
Avima
Chapulobunus
Pseudopachylus
Anoplodactylus
Peripatopsis
Limulus
Scutigera
Eremobates
Synsphyronus
Ixodes
Tetranychus
Daphnia

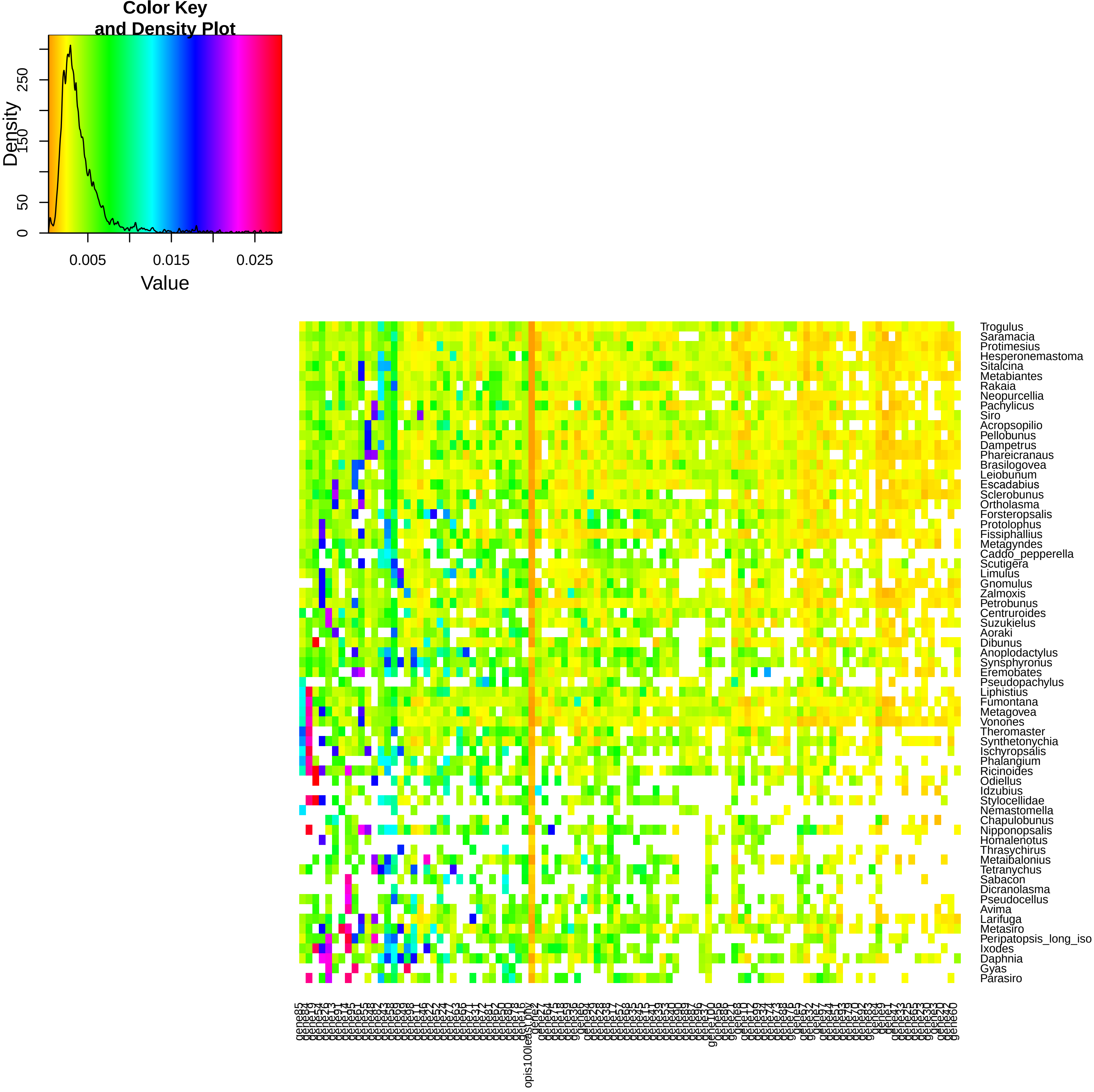
Color Key
and Density Plot
Density
0
50
150
250
0.005
0.015
0.025
Value
Trogulus
Saramacia
Protimesius
Hesperonemastoma
Sitalcina
Metabiantes
Rakaia
Neopurcellia
Pachylicus
Siro
Acropsopilio
Pellobunus
Dampetrus
Phareicranaus
Brasilogovea
Leiobunum
Escadabius
Sclerobunus
Ortholasma
Forsteropsalis
Protolophus
Fissiphallius
Metagyndes
Caddo_pepperella
Scutigera
Limulus
Gnomulus
Zalmoxis
Petrobunus
Centruroides
Suzukielus
Aoraki
Dibunus
Anoplodactylus
Synsphyronus
Eremobates
Pseudopachylus
Liphistius
Fumontana
Metagovea
Vonones
Theromaster
Synthetonychia
Ischyropsalis
Phalangium
Ricinoides
Odiellus
Idzubius
Stylocellidae
Nemastomella
Chapulobunus
Nipponopsalis
Homalenotus
Thrasychirus
Metaibalonius
Tetranychus
Sabacon
Dicranolasma
Pseudocellus
Avima
Larifuga
Metasiro
Peripatopsis_long_iso
Ixodes
Daphnia
Gyas
Parasiro
opis100least.phy

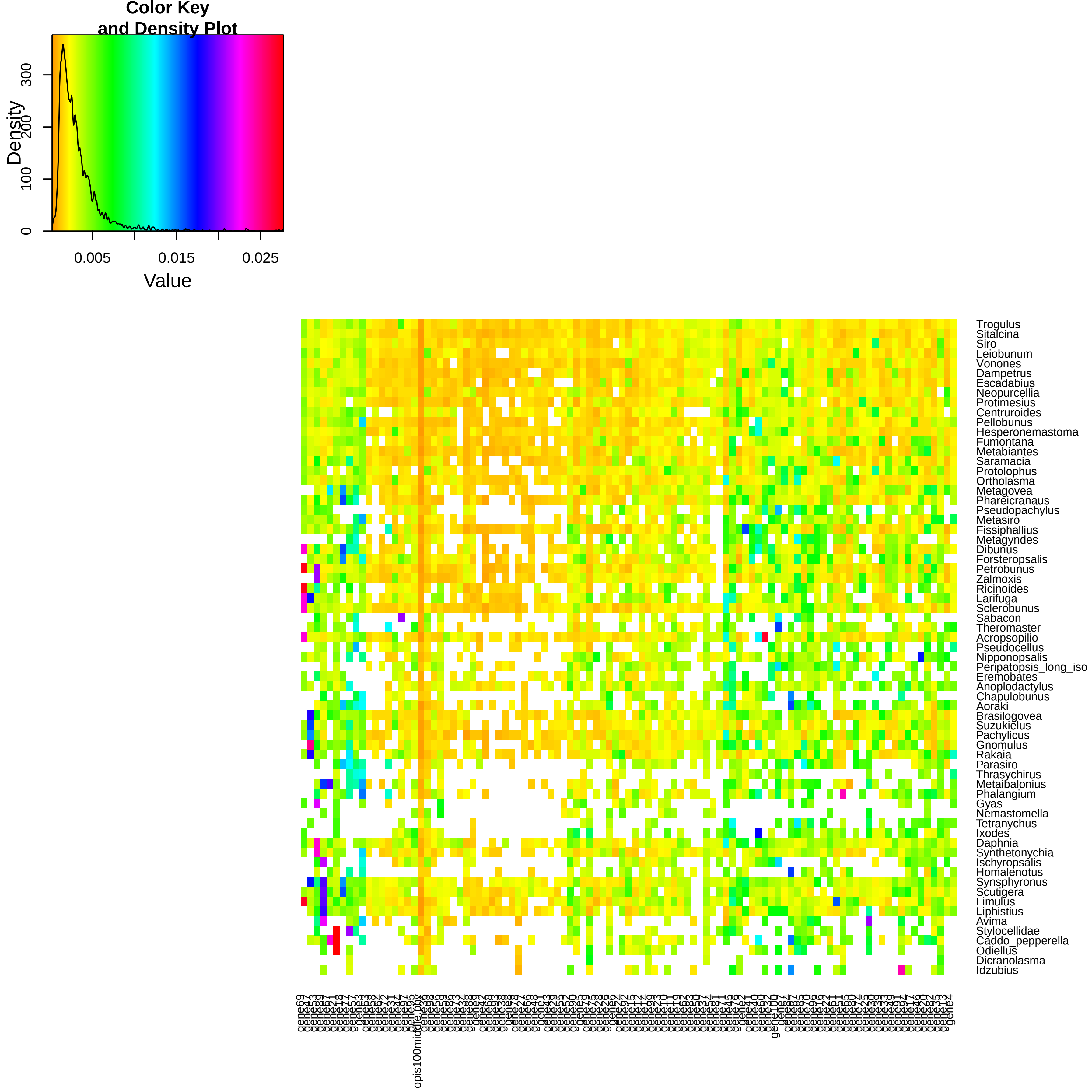
Color Key
and Density Plot
Density
Value
0
100
200
300
0.005
0.015
0.025
Trogulus
Sitalcina
Siro
Leiobunum
Vonones
Dampetrus
Escadabius
Neopurcellia
Protimesius
Centruroides
Pellobunus
Hesperonemastoma
Fumontana
Metabiantes
Saramacia
Protolophus
Ortholasma
Metagovea
Phareicranaus
Pseudopachylus
Metasiro
Fissiphallius
Metagyndes
Dibunus
Forsteropsalis
Petrobunus
Zalmoxis
Ricinoides
Larifuga
Sclerobunus
Sabacon
Theromaster
Acropsopilio
Pseudocellus
Nipponopsalis
Peripatopsis_long_iso
Eremobates
Anoplodactylus
Chapulobunus
Aoraki
Brasilogovea
Suzukielus
Pachylicus
Gnomulus
Rakaia
Parasiro
Thrasychirus
Metaibalonius
Phalangium
Gyas
Nemastomella
Tetranychus
Ixodes
Daphnia
Synthetonychia
Ischyropsalis
Homalenotus
Synsphyronus
Scutigera
Limulus
Liphistius
Avima
Stylocellidae
Caddo_pepperella
Odiellus
Dicranolasma
Idzubius
opis100middle.phy

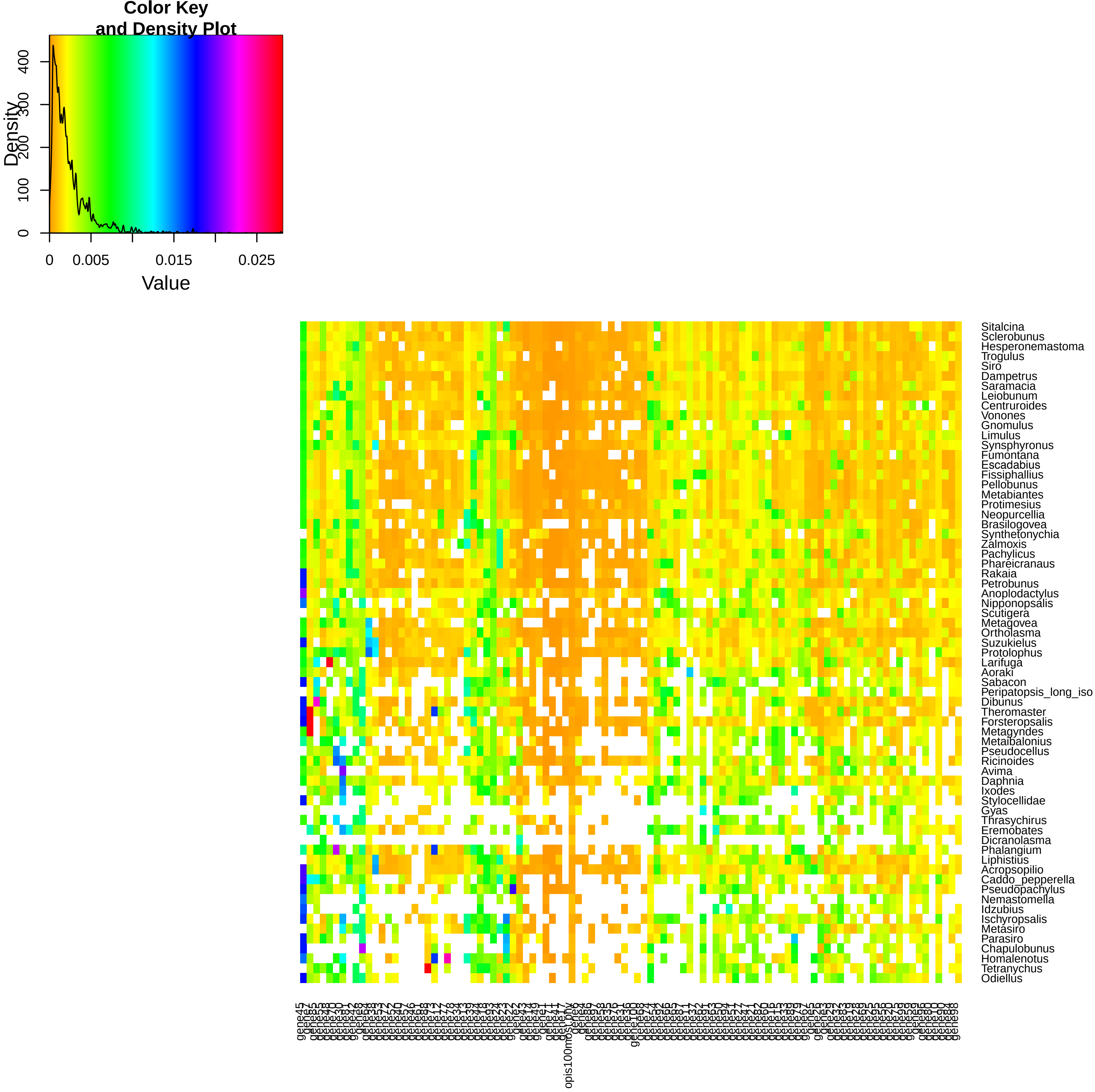

Color Key
and Density Plot
Density
0
100
200
300
400
0
0.005
0.015
0.025
Value
Sitalcina
Sclerobunus
Hesperonemastoma
Trogulus
Siro
Dampetrus
Saramacia
Leiobunum
Centruroides
Vonones
Gnomulus
Limulus
Synsphyronus
Fumontana
Escadabius
Fissiphallius
Pellobunus
Metabiantes
Protimesius
Neopurcellia
Brasilogovea
Synthetonychia
Zalmoxis
Pachylicus
Phareicranaus
Rakaia
Petrobunus
Anoplodactylus
Nipponopsalis
Scutigera
Metagovea
Ortholasma
Suzukielus
Protolophus
Larifuga
Aoraki
Sabacon
Peripatopsis_long_iso
Dibunus
Theromaster
Forsteropsalis
Metagyndes
Metaibalonius
Pseudocellus
Ricinoides
Avima
Daphnia
Ixodes
Stylocellidae
Gyas
Thrasychirus
Eremobates
Dicranolasma
Phalangium
Liphistius
Acropsopilio
Caddo_pepperella
Pseudopachylus
Nemastomella
Idzubius
Ischyropsalis
Metasiro
Parasiro
Chapulobunus
Homalenotus
Tetranychus
Odiellus

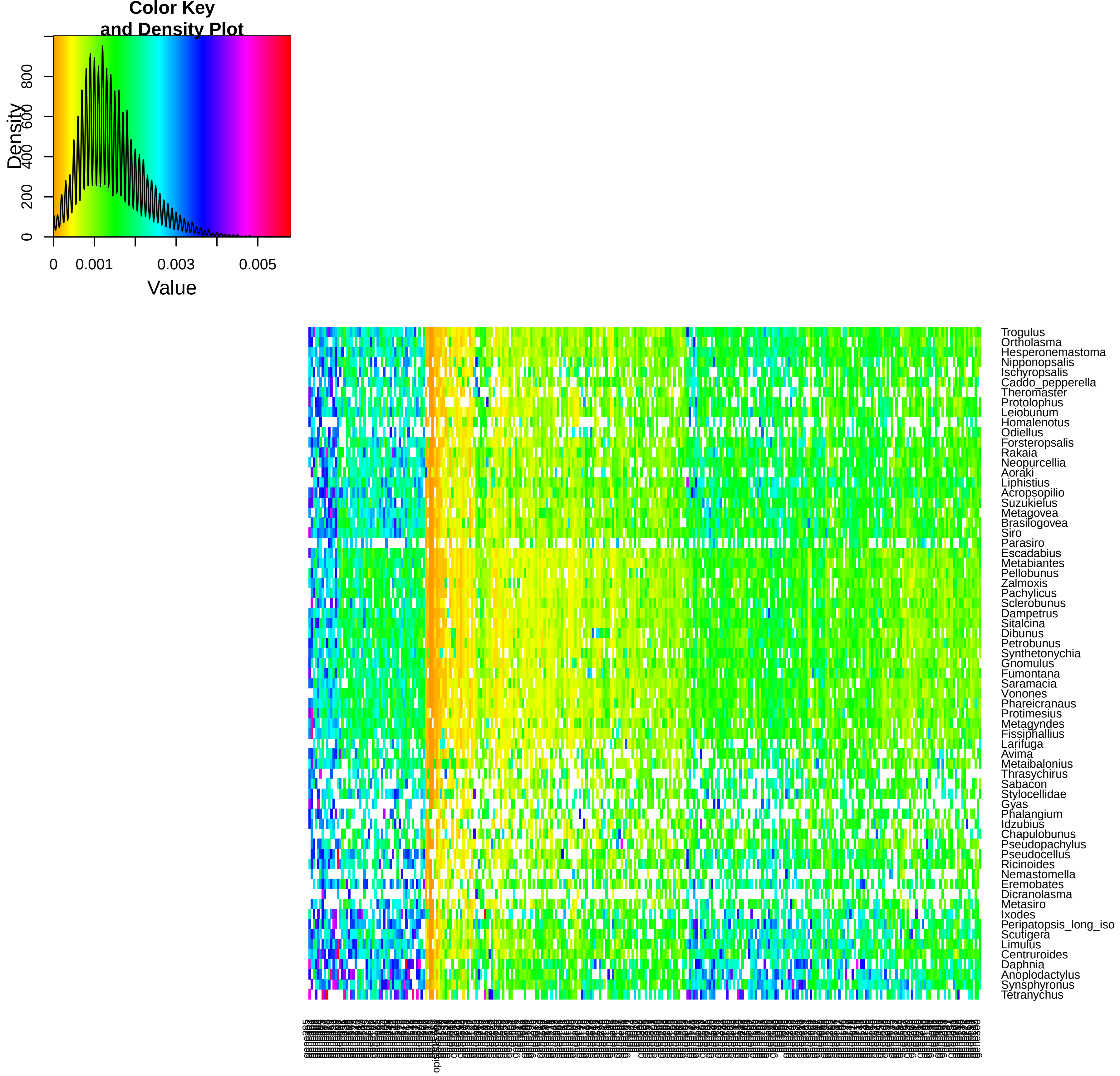
Color Key
and Density Plot
Density
0
200
400
600
800
Value
0
0.001
0.003
0.005
Trogulus
Ortholasma
Hesperonemastoma
Nipponopsalis
Ischyropsalis
Caddo_pepperella
Theromaster
Protolophus
Leiobunum
Homalenotus
Odiellus
Forsteropsalis
Rakaia
Neopurcellia
Aoraki
Liphistius
Acropsopilio
Suzukielus
Metagovea
Brasilogovea
Siro
Parasiro
Escadabius
Metabiantes
Pellobunus
Zalmoxis
Pachylicus
Sclerobunus
Dampetrus
Sitalcina
Dibunus
Petrobunus
Synthetonychia
Gnomulus
Fumontana
Saramacia
Vonones
Phareicranaus
Protimesius
Metagyndes
Fissiphallius
Larifuga
Avima
Metaibalonius
Thrasychirus
Sabacon
Stylocellidae
Gyas
Phalangium
Idzubius
Chapulobunus
Pseudopachylus
Pseudocellus
Ricinoides
Nemastomella
Eremobates
Dicranolasma
Metasiro
Ixodes
Peripatopsis_long_iso
Scutigera
Limulus
Centruroides
Daphnia
Anoplodactylus
Synsphyronus
Tetranychus
