# Supplementary material for: The Opiliones tree of life: shedding light on harvestmen relationships through transcriptomics
Source: Proc Biol Sci. 2017 Feb 22;284(1849):20162340. doi: 10.1098/rspb.2016.2340 (PMC5326524; doi:10.1098/rspb.2016.2340)
Supplement: Table S1 [file rspb20162340supp4.pdf]

Family	Species	MCZ Acc #		SRA #
**CYPHOPHTHALMI**				
Pettalidae	*Aoraki longitarsa*	IZ-137238	http://mczbase.mcz.harvard.edu/guid/MCZ:IZ:137238	SRR5181577
Pettalidae	*Neopurcellia salmoni*	IZ-29310	http://mczbase.mcz.harvard.edu/guid/MCZ:IZ:29310	SRR5181448
Pettalidae	*Rakaia magna australis*	IZ-29212	http://mczbase.mcz.harvard.edu/guid/MCZ:IZ:29212	SRR5181449
Neogoveidae	*Brasilogovea microphaga*	IZ-136559	http://mczbase.mcz.harvard.edu/guid/MCZ:IZ:136559	SRR5185916
Neogoveidae	*Metagovea oviformis*	IZ-136533	http://mczbase.mcz.harvard.edu/guid/MCZ:IZ:136533	SRR5230765
Neogoveidae	*Metasiro savannahensis*	IZ-134557	http://mczbase.mcz.harvard.edu	SRX205297
Stylocellidae	Stylocellidae n.sp.	IZ-140867	http://mczbase.mcz.harvard.edu/guid/MCZ:IZ:140867	SRR5234539
Sironidae	*Parasiro coifatti*	IZ-43732	http://mczbase.mcz.harvard.edu/guid/MCZ:IZ:43732	SRR5235985
Sironidae	*Siro boyerae*	--	Hedin et al. (2014)	SRX450933
Sironidae	*Suzukielus sauteri*	IZ-132257	http://mczbase.mcz.harvard.edu/guid/MCZ:IZ:132257	SRR5234546
**EUPNOI**				
Caddidae	*Caddo pepperella*	IZ-40441	http://mczbase.mcz.harvard.edu/guid/MCZ:IZ:40441	SAMN06309539
Neopilionidae	*Forsteropsalis pureora*	IZ-29216	http://mczbase.mcz.harvard.edu/guid/MCZ:IZ:29216	SRR5234746
Neopilionidae	*Thrasychirus gulosus*	IZ-138161	http://mczbase.mcz.harvard.edu/guid/MCZ:IZ:138161	SRR5234747
Phalangiidae	*Odiellus troguloides*	IZ-43723	http://mczbase.mcz.harvard.edu/guid/MCZ:IZ:43723	SRR5234757
Phalangiidae	*Phalangium opilio*	IZ-29189	http://mczbase.mcz.harvard.edu	SRR1145735
Protolophidae	*Protolophus singularis*	--	Hedin et al. (2014)	SRX450934
Sclerosomatidae	*Gyas titanus*	IZ-45502	http://mczbase.mcz.harvard.edu	SRR5234758
Sclerosomatidae	*Homalenotus remyi*	IZ-43715	http://mczbase.mcz.harvard.edu/guid/MCZ:IZ:43715	SRR5235983
Sclerosomatidae	*Leiobunum verrucosum*	--	Hedin et al. (2014)	SRX450936
**DYSPNOI**				
Acropsopilionidae	*Acropsopilio neozealandiae*	IZ-30457	http://mczbase.mcz.harvard.edu/guid/MCZ:IZ:30457	SRR5235984
Dicranolasmatidae	*Dicranolasma soerenseni*	IZ-43680	http://mczbase.mcz.harvard.edu/guid/MCZ:IZ:43680	SRR5235597
Ischyropsalididae	*Ischyropsalis nodifera*	IZ-45501	http://mczbase.mcz.harvard.edu/guid/MCZ:IZ:45501	SRR5235484
Nemastomatidae	*Ortholasma coronadense*	--	Hedin et al. (2014)	SRX451776
Nemastomatidae	*Nemastomella dubia*	IZ-43691	http://mczbase.mcz.harvard.edu/guid/MCZ:IZ:43691	SRR5235516
Nipponopsalididae	*Nipponopsalis abei*	IZ-45632	http://mczbase.mcz.harvard.edu/guid/MCZ:IZ:45632	SRR5235518
Sabaconidae	*Sabacon cavicolens*	IZ-46883	http://mczbase.mcz.harvard.edu/guid/MCZ:IZ:46883	SAMN06309943
Taracidae	*Hesperonemastoma*	--	Hedin et al. (2014)	SRX450937
Trogulidae	*Trogulus martensi*	--	Hedin et al. (2014)	SRX450964
**LANIATORES/INSIDIATORES**				
Cladonychiidae	*Theromaster sp.*	IZ-46878	http://mczbase.mcz.harvard.edu	SRR5244184
Synthetonychiidae	*Synthetonychia glacialis*	IZ-137212	http://mczbase.mcz.harvard.edu/guid/MCZ:IZ:137212	SRR5236909
Travuniidae	*Sclerobunus robustus*	--	Hedin et al. (2014)	SRX647390
Triaenonychidae	*Larifuga capensis*	IZ-49747	http://mczbase.mcz.harvard.edu/guid/MCZ:IZ:49747	SRR1145742
Triaenonychidae	*Fumontana deprehendor*	IZ-46888	http://mczbase.mcz.harvard.edu/guid/MCZ:IZ:46888	SRR5236916
**LANIATORES/GRASSATORES**				
Agoristenidae	*Avima matintaperera*	IZ-136560	http://mczbase.mcz.harvard.edu	SRR5241472
Assamiidae	*Dampetrus sp.*	IZ-140868	http://mczbase.mcz.harvard.edu/guid/MCZ:IZ:140868	SRR5242745
Biantidae	*Metabiantes sp.*	IZ-49748	http://mczbase.mcz.harvard.edu/guid/MCZ:IZ:49748	SRR1146667
Cosmetidae	*Vonones ornata*	IZ-49750	http://mczbase.mcz.harvard.edu/guid/MCZ:IZ:49750	SRR1145738
Cranaidae	*Phareicranaus manauara*	IZ-136554	http://mczbase.mcz.harvard.edu/guid/MCZ:IZ:136554	SRR5241475
