# Supplementary material for: The Opiliones tree of life: shedding light on harvestmen relationships through transcriptomics
Source: Proc Biol Sci. 2017 Feb 22;284(1849):20162340. doi: 10.1098/rspb.2016.2340 (PMC5326524; doi:10.1098/rspb.2016.2340)
Supplement: Table S2 [file rspb20162340supp5.pdf]

Matrix \ Analysis	PhyML-PCMA	ExaBayes	ExaML	PhyML-IL	PhyloBayes	Indiv. Gene trees for SuperQ	Dating root Opis LN	Dating root Opis UGAM	Dating root Cyphos LN	Dating root Cyphos UGAM	BaCoCa
90% taxon occupancy (78 genes, 13,239 amino ac	X	X	X	X	X	X	X	X	X	X	X
75% taxon occupancy (305 genes, 66,217 amino a	-	X	X	-	X	X	-	-	-	-	X
50% taxon occupancy(1550 genes, 425,716 amino	-	X	X	-	-	X	-	-	-	-	-
100 least conserved genes (21,877 amino acids)	-	X	-	-	X	X	-	-	-	-	X
100 middle genes (29,366 amino acids)	-	-	-	X	X	X	-	-	-	-	X
100 most conserved (26,608 amino acids)	X	-	-	-	X	X	-	-	-	-	X
