# Supplementary material for: The Opiliones tree of life: shedding light on harvestmen relationships through transcriptomics
Source: Proc Biol Sci. 2017 Feb 22;284(1849):20162340. doi: 10.1098/rspb.2016.2340 (PMC5326524; doi:10.1098/rspb.2016.2340)
Supplement: Extended Material and Methods [file rspb20162340supp6.docx]

Extended Material and Methods

(a) Specimens

Specimens of Opiliones selected for this study were collected in a diversity of locales, including major trips to the Brazilian Amazon, Europe, North America and New Zealand, and complemented by smaller trips to other localities and by donations of specimens by colleagues. Taxonomic and geographic details of the represented species and accession codes are available in Table S1; additional information on the specimens can be found in the Museum of Comparative Zoology’s online database MCZbase (<http://mczbase.mcz.harvard.edu>) by using the MCZ accession numbers.

Specimens of Opiliones selected for this study were preserved in RNA*later* and transferred to liquid nitrogen upon arrival to the laboratory and subsequently stored at -80 ºC or, if brought alive to the laboratory, directly flash frozen in liquid nitrogen and preserved in -80 ºC. Total RNA was extracted with TRIzol (Life Sciences) and mRNA was purified with the Dynabeads mRNA Purification Kit (Invitrogen) following manufacturer’s instructions. cDNA libraries were constructed in the Apollo 324 automated system using the PrepX mRNA kit (IntegenX) and sequenced in-house at the FAS Center for Systems Biology in an Illumina Hi-Seq 2500 with a read length of 150 base pairs. Further details about the protocols can be found elsewhere [1, 2].

Our final matrix comprises 54 taxa, including 10 Cyphophthalmi (4 families included; Ogoveidae and Troglosironidae missing), 9 Eupnoi (representatives of all 5 families included), 9 Dyspnoi (all 8 families included), and 26 Laniatores (representatives of 23 families included; missing Gerdesiidae, Guasiniidae, Icaleptidae, Kimulidae, Metasarcidae, Nippononychidae, Pyramidopidae, and Tithaeidae, all families of low diversity and relatively narrow distribution in places difficult to access). As outgroups, we included several chelicerates (the pycnogonid *Anoplodactylus insignis,* the horseshoe crab *Limulus polyphemus,* the pseudoscorpion *Synsphyronus apimelus,* the mite *Tetranychus urticae,* the spider *Liphistius malayanus,* the camel spider *Eremobates* sp*.,* the scorpion *Centruroides sculpturatus,* and the ricinuleids *Ricinoides karschii* and *Pseudocellus pearsei),* one crustacean (*Daphnia pulex*)*,* one myriapod (*Scutigera coleoptrata*) and one onychophoran (*Peripatopsis obervergiensis*) (Table S1).

All raw sequences are deposited in the SRA archive of GenBank under accession numbers specified in Table S1. Data on specimens are available from MCZbase (<http://mczbase.mcz.harvard.edu>).

(b) Orthology assignment and matrix construction

Orthology assignment was based on the OMA algorithm version 0.99.z3 [3], as specified in detail in our previous work [1]. Orthologous genes were aligned with MUSCLE [4]. Probabilistic alignment masking of each orthologous gene was done with ZORRO [5], and positions below a confidence threshold of 5 [see 1] were removed from the alignments.

A first approach to matrix construction based on gene occupancy was followed [6]. These include matrices with >90%, >75% and 50% gene occupancy (a gene occupancy of 50% means that an OMA orthologous group is selected if present in at least 50% of the taxa), resulting in three matrices with 78 genes (matrix I), 305 genes (matrix II) and 1,550 genes (matrix III), respectively.

Additionally we explored subsets of 100 genes with different evolutionary rates (measured as percentage of identical sites), as in Fernández et al. [7]. From the 1,550-gene matrix, we selected the 100 least conserved genes (matrix IV), the 100 that had an evolutionary rate closest to the mean observed in this data set (varying from 27.9 to 28.4% of identical sites; matrix V), and the 100 most conserved genes (matrix VI). Information about the number of amino acids and the percentage of missing data for each matrix can be found in Table S2.

(c) Phylogenetic analyses

Maximum likelihood inference was conducted with PhyML-PCMA [8], ExaML [9] and PhyML v.3.0.3 implementing the integrated branch length flag (i.e., this approach integrates branch length over a wide range of scenarios, therefore allowing implementation of a further correction of heterotachy not considered by mixture models). Bootstrap support values were estimated with 100 replicates under the rapid bootstrapping algorithm [10]. In PhyML-PCMA, we selected 20 principal components and empirical amino acid frequencies. The per site rate category model was selected in ExaML.

Bayesian analyses were conducted with ExaBayes [11] and PhyloBayes MPI 1.4e [12] using the site-heterogeneous CAT-GTR model of evolution in the latter software [13]. Two independent Markov chain Monte Carlo (MCMC) chains were run for > 5,000 cycles. The initial 20% trees sampled in each MCMC run prior to convergence (i.e., when maximum bipartition discrepancies across chains < 0.1) were discarded as the burn-in. A 50% majority-rule consensus tree was then computed from the remaining trees sampled every 10 cycles.

Compositional homogeneity of each gene and taxon was evaluated in BaCoCa [14]. The relative composition frequency variability (RCFV) values (that measures the absolute deviation from the mean for each amino acid for each taxon) was plotted in a heatmap using the R package gplots with an R script modified from Kück and Struck [14]. None of the genes in any of the matrices showed any signs of compositional heterogeneity (i.e., RCFV values were lower than 0.025), therefore there was no need to eliminate them from our matrices (figure S2).

Due to computational constraints, and in order to improve the efficiency of our analyses, not all analyses were run for all the matrices (see figures 2, S1).

(d) Molecular dating

The fossil record of Opiliones is well documented, and most key fossils have been included in prior phylogenetic analyses, making their placement in a phylogenetic context precise. We mostly follow the strategy and fossil placement of Sharma and Giribet [15], who conducted tip dating in one of their analyses. The oldest Opiliones fossil, *Eophalangium sheari*, from the Early Devonian Rhynie Cherts [16, 17] is now interpreted as a member of the extant suborder Tetrophthalmi, together with *Hastocularis argus* from the Carboniferous Montceau-les-Mines Lagerstätte [18]. Tetrophthalmi is the putative sister group of Cyphophthalmi [15]. *Eophalangium* had been originally interpreted as a stem Eupnoi, but now we use it as a minimum age for the origin of its sister group, Cyphophthalmi, which here corresponds to the node splitting Cyphophthalmi from Phalangida. Given how close this fossil is to the root of Opiliones we use a soft bound of 411 Ma for the floor of Opiliones. A second analysis was conducted considering the age of *Eophalangium* as the minimum age of Cyphophthalmi, based on the rationale explained above.

Two other key Carboniferous fossils [19] are used to constrain the non-caddid Eupnoi (based on the modern-looking *Macrogyion cronus*) and the non-acropsopilionid Dyspnoi (based on *Ameticos scolos*), and applied soft bounds of 305 Ma to each of these clades.

Additional Eupnoi fossils from the Middle Jurassic (approx. 165 Mya) of Daohugou, Inner Mongolia, China are known [20], and one species, *Mesobunus dunlopi*, preserved the penis, allowing placement in Sclerosomatidae [21], and constraining the corresponding node to 165 Ma.

Other relevant fossils include Upper Cretaceous (lowermost Cenomanian, ca. 99 Ma) Burmese amber from Myanmar [22, 23]. *Petrobunoides sharmai* (Selden et al. 2016), placed in the extant family Epedanidae, was used as a constrain for the superfamily Epedanoidea. *Halitherses grimaldii* has been recently reinterpreted as a new family of uncertain affinities [24, 25], and thus is of little help for dating, as an older fossil was already used to constrain the non-Acropsopilionidae Dyspnoi. *Paleosiro burmanicum* was originally described as a member of the Cyphophthalmi family Sironidae [23], but it is of stylocellid affinities [26]. Given the uncertain placement of the fossil within Stylocellidae and the presence of a single stylocellid terminal, we do not use it for node dating to avoid a “push towards the present” effect [see 27].

The split between Onychophora and Arthropoda was used to root the tree with a uniform prior of 528–558 Ma. The split between Xiphosura and Arachnida was constrained with a uniform prior of 465–485 Ma, based on the age of *Lunataspis aurora*. *Proscorpius osborni* was selected as being an anatomically well-understood Silurian scorpion (Whitfield 1885), setting a constraint on the split of Scorpiones from Tetrapulmonata.

Divergence dates were estimated using the Bayesian relaxed molecular clock approach as implemented in PhyloBayes v. 3.3f [12] under the autocorrelated lognormal and uncorrelated gamma multipliers models, resulting in four analyses (i.e., these two models were applied to both calibration configurations described above, with the age of *Eophalangium* as the minimum age of Cyphophthalmi or as the floor of Opiliones). Two independent MCMC chains were run for each analysis (10,000–12,000 cycles). The calibration constraints were used with soft bounds [28] under a birth–death prior.

References

1. Fernández R, Laumer, CE, Vahtera, V, Libro, S, Kaluziak, S, Sharma, PP, Pérez-Porro, AR, Edgecombe, GD, Giribet, G. 2014 Evaluating topological conflict in centipede phylogeny using transcriptomic data sets. *Mol. Biol. Evol.* **31**, 1500-1513. (doi:10.1093/molbev/msu108)

2. Fernández R, Edgecombe, GD, Giribet, G. 2016 Exploring phylogenetic relationships within Myriapoda and the effects of matrix composition and occupancy on phylogenomic reconstruction. *Syst. Biol.* **65**, 871-889. (doi:10.1093/sysbio/syw041)

3. Altenhoff AM, Gil, M, Gonnet, GH, Dessimoz, C. 2013 Inferring hierarchical orthologous groups from orthologous gene pairs. *PLoS One* **8**, e53786. (doi:10.1371/journal.pone.0053786)

4. Edgar RC. 2004 MUSCLE: multiple sequence alignment with high accuracy and high throughput. *Nucleic Acids Res.* **32**, 1792-1797.

5. Wu M, Chatterji, S, Eisen, JA. 2012 Accounting for alignment uncertainty in phylogenomics. *PLoS One* **7**, e30288. (doi:10.1371/journal.pone.0030288)

6. Hejnol A, Obst, M, Stamatakis, A, M., O, Rouse, GW, Edgecombe, GD, Martinez, P, Baguñà, J, Bailly, X, Jondelius, U *et al.* 2009 Assessing the root of bilaterian animals with scalable phylogenomic methods. *Proc. R. Soc. B Biol. Sci.* **276**, 4261-4270. (doi:10.1098/rspb.2009.0896)

7. Fernández R, Hormiga, G, Giribet, G. 2014 Phylogenomic analysis of spiders reveals nonmonophyly of orb weavers. *Curr. Biol.* **24**, 1772-1777. (doi:10.1016/j.cub.2014.06.035)

8. Zoller S, Schneider, A. 2013 Improving phylogenetic inference with a semiempirical amino acid substitution model. *Mol. Biol. Evol.* **30**, 469-479. (doi:10.1093/molbev/mss229)

9. Aberer AJ, Stamatakis, A. 2013 ExaML: Exascale Maximum Likelihood: Program and documentation available at: <http://sco.h-its.org/exelixis/web/software/examl/index.html>.

10. Stamatakis A, Hoover, P, Rougemont, J. 2008 A rapid bootstrap algorithm for the RAxML Web servers. *Syst. Biol.* **57**, 758-771. (doi:10.1080/10635150802429642)

11. Aberer AJ, Kobert, K, Stamatakis, A. 2014 ExaBayes: massively parallel Bayesian tree inference for the whole-genome era. *Mol. Biol. Evol.* **31**, 2553-2556. (doi:10.1093/molbev/msu236)

12. Lartillot N, Rodrigue, N, Stubbs, D, Richer, J. 2013 PhyloBayes MPI: Phylogenetic reconstruction with infinite mixtures of profiles in a parallel environment. *Syst. Biol.* **62**, 611-615. (doi:10.1093/Sysbio/Syt022)

13. Lartillot N, Philippe, H. 2004 A Bayesian mixture model for across-site heterogeneities in the amino-acid replacement process. *Mol. Biol. Evol.* **21**, 1095-1109. (doi:10.1093/molbev/msh112)

14. Kück P, Struck, TH. 2014 BaCoCa – A heuristic software tool for the parallel assessment of sequence biases in hundreds of gene and taxon partitions. *Mol. Phylogenet. Evol.* **70**, 94-98. (doi:10.1016/j.ympev.2013.09.011)

15. Sharma PP, Giribet, G. 2014 A revised dated phylogeny of the arachnid order Opiliones. *Front. Genet.* **5**, 255. (doi:10.3389/fgene.2014.00255)

16. Dunlop JA, Anderson, LI, Kerp, H, Hass, H. 2003 Preserved organs of Devonian harvestmen. *Nature* **425**, 916.

17. Dunlop JA, Anderson, LI, Kerp, H, Hass, H. 2004 A harvestman (Arachnida: Opiliones) from the Early Devonian Rhynie cherts, Aberdeenshire, Scotland. *Trans. r. Soc. Edinburgh, Earth Sci.* **94**, 341-354.

18. Garwood RJ, Sharma, PP, Dunlop, JA, Giribet, G. 2014 A new stem-group Palaeozoic harvestman revealed through integration of phylogenetics and development. *Curr. Biol.* **24**, 1-7. (doi:10.1016/j.cub.2014.03.039)

19. Garwood RJ, Dunlop, JA, Giribet, G, Sutton, MD. 2011 Anatomically modern Carboniferous harvestmen demonstrate early cladogenesis and stasis in Opiliones. *Nat. Commun.* **2**, 444. (doi:10.1038/ncomms1458)

20. Huang D, Selden, PA, Dunlop, JA. 2009 Harvestmen (Arachnida: Opiliones) from the Middle Jurassic of China. *Naturwissenchaften* **96**, 955-962. (doi:10.1007/s00114-009-0556-3)

21. Giribet G, Tourinho, AL, Shih, C, Ren, D. 2012 An exquisitely preserved harvestman (Arthropoda, Arachnida, Opiliones) from the Middle Jurassic of China. *Organisms, Diversity & Evolution* **12**, 51-56. (doi:10.1007/s13127-011-0067-x)

22. Giribet G, Dunlop, JA. 2005 First identifiable Mesozoic harvestman (Opiliones: Dyspnoi) from Cretaceous Burmese amber. *Proc. Biol. Sci.* **272**, 1007-1013.

23. Poinar G. 2008 *Palaeosiro burmanicum* n. gen., n. sp., a fossil Cyphophthalmi (Arachnida: Opiliones: Sironidae) in Early Cretaceous Burmese amber. In *Advances in Arachnology and Developmental Biology. Papers dedicated to Prof. Dr. Bozidar Curcic*, vol. Monographs, 12 (ed. S E Makarov, R N Dimitrijevic), pp. 267-274. Vienna, Belgrade, Sofia: Faculty of Life Sciences, University of Vienna, and Serbian Academy of Sciences and Arts.

24. Dunlop JA, Selden, PA, Giribet, G. 2016 Penis morphology in a Burmese amber harvestman. *Sci. Nat.* **103**, 11. (doi:10.1007/s00114-016-1337-4)

25. Shear WA. 2010 New species and records of ortholasmatine harvestmen from México, Honduras, and the western United States (Opiliones, Nemastomatidae, Ortholasmatinae). *ZooKeys* **52**, 9-45.

26. Giribet G, Sharma, PP, Benavides, LR, Boyer, SL, Clouse, RM, de Bivort, BL, Dimitrov, D, Kawauchi, GY, Murienne, JY, Schwendinger, PJ. 2012 Evolutionary and biogeographical history of an ancient and global group of arachnids (Arachnida: Opiliones: Cyphophthalmi) with a new taxonomic arrangement. *Biol. J. Linn. Soc.* **105**, 92-130.

27. Giribet G. 2015 Morphology should not be forgotten in the era of genomics—a phylogenetic perspective. *Zool. Anz.* **256**, 96-103. (doi:10.1016/j.jcz.2015.01.003)

28. Yang ZH, Rannala, B. 2006 Bayesian estimation of species divergence times under a molecular clock using multiple fossil calibrations with soft bounds. *Mol. Biol. Evol.* **23**, 212-226. (doi:10.1093/molbev/msj024)
